# Editorial: Protein Degradation, Aggregation, Membrane Trafficking and Exosomes in Neuronal Health and Disease

**DOI:** 10.3389/fnmol.2022.958507

**Published:** 2022-06-23

**Authors:** Douglas S. Campbell, Miguel Díaz-Hernández, Beatriz Alvarez-Castelao

**Affiliations:** ^1^Graduate School of Pharmaceutical Sciences, Kyoto University, Kyoto, Japan; ^2^Biochemistry and Molecular Biology Department, Veterinary School, Complutense University of Madrid, Madrid, Spain

**Keywords:** degradation, neurodegeneration, exosomes, disease, membrane, neuron

Neurons are highly specialized and polarized cells. Comprising usually of a single long axon and multiple shorter dendrites, their unique geometry creates challenges for cellular homeostasis. For example, how and where cellular components are expressed or removed at the right time and place, or if any abnormality in neurons or other neural-system cells may compromise their function and initiate disease pathology are questions that remain largely unanswered. This Research Topic aims to cover the beforementioned questions with new discoveries but also revising the preexisting literature. We as editors, and the authors of the articles tackle main processes of neuronal cell biology homeostasis, such as protein degradation, membrane trafficking, or exosomes and explore some of their normal and abnormal roles, therefore this topic is covering fundamental aspects of neuronal cell biology, which are related to disease.

One important pathway for protein degradation is the ubiquitin proteasome system (UPS), which plays key roles in different physiological processes, such as fear conditioning, a type of memory implicated in several brain diseases. Despite key roles for the UPS in fear conditioning and the possibility it could underlie post-traumatic stress disorder (PTSD), a systematic screening for substrates of this pathway had not been performed and proteasome targets had remained unknown. Here, Farrell et al., *via* proteomics and liquid chromatography/mass spectrometry (LC/MS) describe proteasome targets in the amygdala following fear conditioning, and find evidence that these targets can vary depending on the sex. The authors raise the possibility that despite the targets being different between sexes [they have previously shown differences in a role for the UPS in fear conditioning between male and female mice (Martin et al., 2021)], the underlying cellular expression of the fear conditioning, such as structural remodeling of neurons could be similar between the sexes.

As we learn more about neurological diseases, more complexity is unearthed and interactions between cellular systems are revealed. Initial studies identified alterations in proteostasis including protein aggregation underlying disorders such as Alzheimer's disease (AD) and others. However, clinical trials with agents that attempt to remove aggregated proteins have unfortunately largely failed to provide a significant delay in disease progression or improve symptoms. While there can be many reasons for these failures, one could be the failure of several cellular systems interacting together. Therefore, effective therapies may be needed which target multiple cellular pathways. Recently, the identification of mediators of inflammation in neurological diseases provides a system which may interact with proteostasis.

Ebstein et al., review the importance of UPS substrates in neurodevelopmental disorders. In their survey they found that many of these protein substrates have roles in the innate immune response, establishing the possible involvement of chronic inflammation in these disorders. In a similar direction Hulse and Bhaskar, discuss the crosstalk between the aggregation prone proteins normally involved in Alzheimer's and Parkinson's diseases (AD/PD) and the multi-protein inflammasome complex (ASC). Instead of being a byproduct of protein aggregation, inflammation mediated by the NLRP3 inflammasome and ASC can act as a driver stabilizing a feedback loop that promotes neurodegeneration. Release of ASC and cytokines extracellularly by microglia and subsequent uptake of ASC by other microglia propagates the spreading of protein aggregation in the brain.

Autophagosomes and endosomes transport proteins from the synaptic connections to the soma for its degradation, this is an incredibly demanding event, thus being susceptible to be involved in pathological conditions, Hu et al., review the critical role of the neuronal endolysosomal trafficking pathway in ischemic brain injury. Another type of vesicle, the extracellular vesicles (EVs) are critical for cell-to-cell communication, their cargo is highly varied including proteins, nucleic acids and mitochondria, Amari and Germain carefully reviewed and discussed recent evidence suggesting that mitochondria transferred through EVs have the capacity to alter metabolic and inflammatory responses in the recipient cells. Besides the capacity of EVs to elicit a response in the target cells, EVs can also accelerate the spreading of protein aggregation, and this capacity is closely related to lipid alterations. Estes et al., examined how these two pathways accelerate the progression of both AD and PD. A better understanding of EVs, and Lipid metabolic pathways and how they contribute to the spread of protein aggregation could lead to the discovery of new therapeutic targets to slow the spread down. One known cargo of EVs is a-Synuclein, which is related to PD. Paralleled to motor dysfunction there is an incremental increase in exosomal a-synuclein levels in plasma. This increase could be used to study PD progression or even help its diagnosis, for this reason it is important to understand how a-synuclein reaches the exosomes. Sepulveda et al., evaluate the relationship between the autophagy-lysosomal pathway and its contribution to the secretion of a-synuclein by exosomes. Some neurodegenerative disorders such as Charcot-Marie-Tooth (CMT) or distal hereditary motor neuropathy arise as consequences of mutations in the heat shock protein 27 (HSP27). However, the complete structure of this protein remains to be fully understood, Holguin et al., review recent insights providing the required structural context to explain the relationship between mutations in HSP27 and the resulting loss or gain of function that leads to disease. The discovery of new genes related to brain diseases is crucial to understanding the pathological processes and to developing new treatments. Chuvakova et al., studied groups of genes related to Audiogenic Epilepsy (AE). Comparing transcriptomes from the *corpora quadrigemina* in the midbrain zone-a region crucial for AE development-between three rat strains with different propensities to suffer AE. The Krushinky-Moldodkina strain, most prone to AE showed increased expression of genes involved in the positive regulation of the MAPK signaling cascade and genes involved in the positive regulation of apoptotic processes, among others. These data show the complex multigenic nature of AE inheritance in rodents. The maintenance of proteostasis is critical to the physiological functioning of neurons, abnormalities in which can play roles from neurodegeneration to addiction. Addiction propensity or risk is related to changes in proteostasis *via* endoplasmic-reticulum-associated degradation (ERAD), with micro RNA (miRNA) regulation playing an important role. In this topic Wang et al. describe that miR-181a may be indirectly responsible for methamphetamine addiction by down-regulating GABAAa1 through the regulation of ERAD, rather than through direct regulation of GABAAa1 itself. Furthermore, low-dose methamphetamine appears to be neuroprotective in other conditions where changes in proteostasis and ERAD occur, such as Alzheimer's amyloid pathology via the down regulation of miR-181a. Further studies could implicate miR-181a as an additional strategy for the treatment of addiction and neurodegenerative disorders.

## Author Contributions

DSC and BA-C wrote the manuscript. MD-H revised the manuscript. All authors contributed to the article and approved the submitted version.

## Funding

DSC was funded by German Research Foundation (DFG) Research Grant (CA 1530/1-1), Japanese Society for the Promotion of Science (JSPS) Grants in aid (Kakenhi) for Research Startup (18H06093, 19K21215), and Grant in aid (Kakenhi) for Basic Research (C) (20K06902). MD-H was funded by Spanish Ministry of Economy and Competitiveness RTI2018-095753-B-I00, European Union project H2020-MSCA-ITN-2017 number 766124, and UCM-Santander Central Hispano BankPR41/17-21014. BA-C is funded by the Spanish Ministry of Science and innovation (Ramón y Cajal- RYC2018-024435-I and PID2020-113270RA-I00) and by the Autonomous Community of Madrid (Atracción de Talento-2019T1/BMD-14057) grants.

## Conflict of Interest

The authors declare that the research was conducted in the absence of any commercial or financial relationships that could be construed as a potential conflict of interest.

## Publisher's Note

All claims expressed in this article are solely those of the authors and do not necessarily represent those of their affiliated organizations, or those of the publisher, the editors and the reviewers. Any product that may be evaluated in this article, or claim that may be made by its manufacturer, is not guaranteed or endorsed by the publisher.
